# The role of fluid friction in streamer formation and biofilm growth

**DOI:** 10.1038/s41522-024-00633-2

**Published:** 2025-01-15

**Authors:** Cornelius Wittig, Michael Wagner, Romain Vallon, Thomas Crouzier, Wouter van der Wijngaart, Harald Horn, Shervin Bagheri

**Affiliations:** 1 FLOW, Department of Engineering Mechanics, KTH, Stockholm, Sweden; 2 Institute of Biological Interfaces (IBG-1), Karlsruhe Institute of Technology, Eggenstein-Leopoldshafen, Germany; 3 Department of Health Technology, DTU, Kongens Lyngby, Denmark; 4 Division of Micro and Nanosystems, Department of Intelligent Systems, KTH, Stockholm, Sweden; 5 Engler-Bunte-Institut, Karlsruhe Institute of Technology, Water Chemistry and Water Technology, Karlsruhe, Germany

**Keywords:** Biofilms, Water microbiology

## Abstract

Biofilms constitute one of the most common forms of living matter, playing an increasingly important role in technology, health, and ecology. While it is well established that biofilm growth and morphology are highly dependent on the external flow environment, the precise role of fluid friction has remained elusive. We grew Bacillus subtilis biofilms on flat surfaces of a channel in a laminar flow at wall shear stresses spanning one order of magnitude (*τ*_*w*_ = 0.068 Pa to *τ*_*w*_ = 0.67 Pa). By monitoring the three-dimensional distribution of biofilm over seven days, we found that the biofilms consist of smaller microcolonies, shaped like leaning pillars, many of which feature a streamer in the form of a thin filament that originates near the tip of the pillar. While the shape, size, and distribution of these microcolonies depend on the imposed shear stress, the same structural features appear consistently for all shear stress values. The formation of streamers occurs after the development of a base structure, suggesting that the latter induces a secondary flow that triggers streamer formation. Moreover, we observed that the biofilm volume grows approximately linearly over seven days for all shear stress values, with a growth rate inversely proportional to the wall shear stress. We develop a scaling model, providing insight into the mechanisms by which friction limits biofilm growth.

## Introduction

Bacteria suspended in a matrix of extracellular polymeric substances (EPS) are known as biofilms and, given enough time, grow on nearly all engineered surfaces in aquatic environments. In a biofilm, EPS serves to protect the bacteria from biological, chemical, and mechanical stress^1,2^. The resulting biofilm may be described as a growing, viscoelastic material^3^. In natural settings and most applications, the external environment for biofilms is a flowing fluid, which serves as a means of delivery of nutrients, oxygen and other substances necessary for the biofilm to survive. However, the flow also imposes forces on the biofilm. These forces shape the biofilm through processes such as erosion, sloughing, and other fluid-structure interactions.

In the presence of flow, relatively thin filamentous structures called streamers are often observed. These streamers have been associated with increased flow resistance due to enhanced surface friction^4^ and their oscillating motion at high Reynolds numbers^5,6^. Early studies detected streamers in turbulent flows^5–7^, where the biofilm microcolonies consisted of an attached base with a filament that extended downstream. These experiments measured the length of a streamer while varying the shear stress to deduce the deformation of the streamer and, thus also, its rheological properties. While these studies were instrumental in establishing similarities between biofilms and complex fluids, the focus was not on the mechanisms responsible for the streamer formation. Later experiments used microfluidic devices to obtain insight into the formation process of streamers behind corners of a tortuous channel^8,9^, in porous media^10–12^ and behind individual cylinders^4^. These studies proposed that “secondary fluid motion” promote streamer development. Here, secondary motion refers to viscous flow that undergoes spatial modulation as a result of a physical obstruction, for example, a corner^4,8,13^. It has been shown that secondary motions in the vicinity of an obstruction concentrate cells and thus promote a build-up of biomass at the wall^14^. This build-up occurs continuously, forming an expanding cluster of biofilm. The increase in local shear stress at the obstruction can fluidise the biofilm and stretch it into a filament. Here, fluidisation refers to a purely viscous response of the biofilm^6^. The streamer then extends beyond the obstruction, until it reaches a region where the local shear stress on the streamer is below the critical threshold for fluidisation^14^.

In addition to influencing the formation of specific structures in biofilms, external flows also affect the rate at which biofilms expand. Horn and Hempel^15^ investigated biofilm accumulation under constant shear stress in a circular pipe, observing nearly linear growth over 108 days. Bakke et al.^16^ observed a similar trend in square ducts over 13 days. Gierl et al.^17^ confirmed these observations for *Bacillus subtilis* using a robotic platform to monitor biofilm growth in multiple flow cells over 6 days.

For most species, higher fluid shear stresses have been shown to reduce biomass and result in more compact formations^18–21^. Paul et al.^22^ reported an exponential decline in the thickness of mature biofilms as wall shear stress increased from *τ*_*w*_ = 0.3 Pa to *τ*_*w*_ = 13 Pa. Similarly, Chun et al.^18^ investigated the very early stages of biofilm growth (4 h) and demonstrated a linear decrease in thickness, mass and coverage for *Cobetia marina* and *Pseudonomas aeruginosa* with increasing shear stress (*τ*_*w*_ = 0.2 Pa to *τ*_*w*_ = 5.6 Pa). Interestingly, Chang et al.^23^ found that increasing wall shear stress from 0.23 Pa to 0.68 Pa led to an increase in the average biofilm thickness for *Bacillus sp*., which they attributed to enhanced EPS production under these specific conditions. However, at higher wall shear stresses, their study also observed a reduction in biofilm volume.

Note that these previous studies have either investigated biofilm growth over time at a single value of shear stress^15–17^ or taken end-point measurements at varying wall-shear stress values^18,22^. Building on the robotic platform presented by Gierl et al.^17^, we present the first experimental setup enabling the longitudinal study of biofilms under varying levels of wall shear stress over several days. We measure the growth of *Bacillus subtilis* (NCIB 3610)—the same strain used by Gierl et al.^17^—biofilms over 1 week. Three-dimensional scans of these biofilms are acquired in situ using optical coherence tomography (OCT). We consistently observe streamers on biofilm structures growing on smooth, flat walls. In contrast to earlier observations of streamers in turbulent flows and in microfluidic devices with geometric defects, the laminar channel flow studied here does not exhibit any secondary flows. However, our data suggests that the observed streamers are related to a secondary motion induced by the biofilm itself. These secondary flows enable the capture of floating cells and aggregates in accordance with the theory proposed by Rusconi et al.^8^. To the best of our knowledge, our time-resolved measurement of the dependency of biofilm growth on shear stress (spanning one order of magnitude in size) is the first of its kind. By analysing this data, we develop a qualitative model of friction-limited growth that agrees with our experimental observations, providing an explicit dependence of biovolume on wall shear stress and time.

Our study has implications for applications where the prediction of biofilm accumulation on surfaces exposed to fluid flows is crucial, including marine infrastructure, food processing, and medical devices. In particular, the prediction of the drag increase of fouled ship hulls has been a subject of intensive research for many years. Previous studies have investigated the drag production by (patchy) biofilms^24–26^, and biofilm streamers^5,27^. The fuel costs due to fouling are considerable. For example, slime fouling can increase the required shaft power of a ship by up to 18%^28^. It is our hope that this work will contribute to better models for predicting the early stages of biofouling.

## Results

### Biofilm morphology

**Fig. 1:**
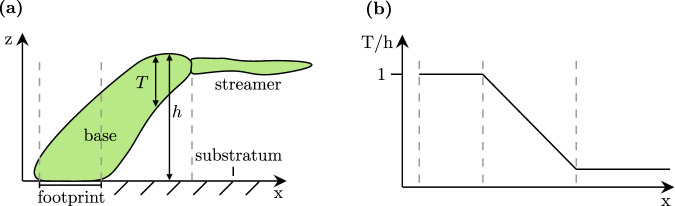
Schematic structure of a microcolony. **a** illustrates two measures of the vertical extent of the biofilm: (1) Biofilm height (*h*), which includes any empty spaces, and (2) Biofilm thickness (*T*), which excludes voids. **b** Schematic of how the solidity measure *T*/*h* changes along one biofilm structure. Vertical dashed lines in (**a**) and (**b**) correspond to the same location.

We begin by examining how biofilm morphology evolves over time for different levels of wall shear stress. We grew *Bacillus subtilis* biofilm in a laminar flow channel (length 70 mm, width 20 mm, height 1–2 mm) at six different levels of wall shear stress. Over the course of 7 days, we extracted 3D spatial measurements of the biofilm using optical coherence tomography (OCT). The experimental conditions are summarised in Table 1, with further details provided in the “Methods”. Using these measurements, the vertical extent of the biofilm can be characterised by two different measures, as defined in Fig. 1a. The biofilm height, *h*, refers to the distance from the substratum to the highest point containing biofilm. The biofilm thickness $T(x,y,t)=\mathop{\int}\nolimits_{0}^{h}b(x,y,z,t)\,{\rm{d}}z$ is defined as the height of the biofilm excluding any voids, characterising the amount of biofilm at each position. Here, *b* represents the binarised volumetric biofilm data.

**Table 1:** Parameters of cases 1–6

Case			1	2	3	4	5	6
Channel Height	*H*	[mm]	2	$\sqrt{2}$	2	1	$\sqrt{2}$	1
Hydraulic diameter	*D*_*H*_	[mm]	3.63	2.64	3.63	2.64	1.9	1.9
Flow rate	*Q*	[mL s^−1^]	1	1	2.63	1	2.63	2.63
Viscosity	*μ*	[mPa s]	0.91	0.91	0.85	0.91	0.85	0.85
Reynolds number	*Re*	[-]	100	100	300	100	300	300
Wall shear stress	*τ*_*w*_	[Pa]	0.068	0.14	0.17	0.27	0.34	0.67

The cases are sorted by increasing *τ*_*w*_.

#### Development of a sample biofilm in time

**Fig. 2:**
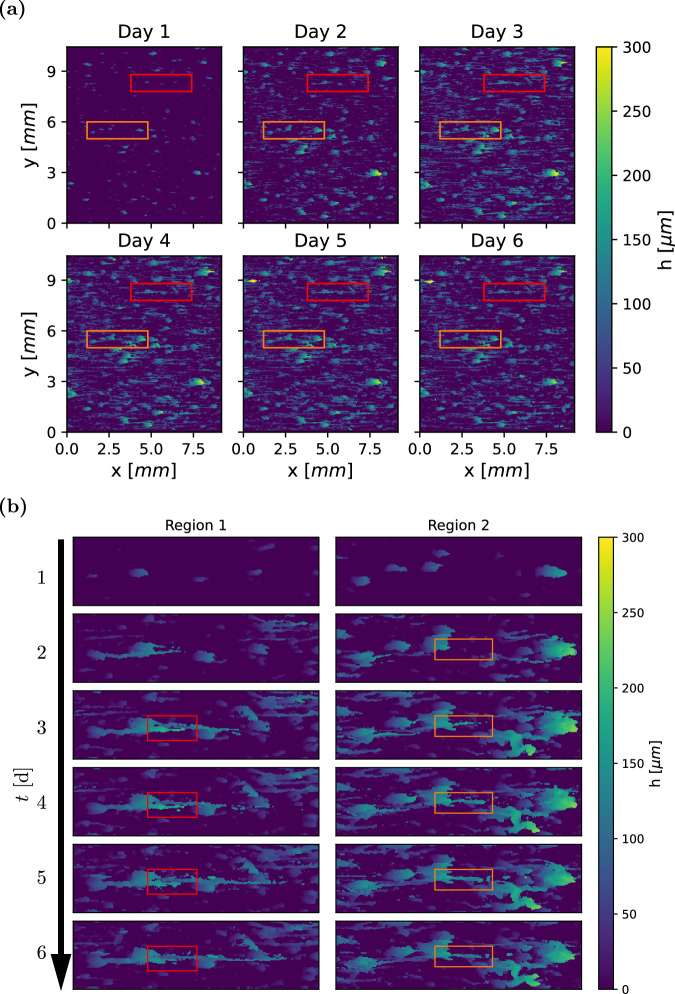
Biofilm growth and streamer development. Case 1, corresponding to *τ*_*w*_ = 0.068 Pa. The flow direction is from left to right. Frame **a** shows changes in biofilm height *h* over one week, where individual biofilm structures grow progressively, and new structures emerge. Frame **b** focuses on streamer development. Region 1 and 2 correspond to the red and orange rectangles in (**a**), respectively. Streamer formation begins on the second day, with the boxes tracing the development of a representative streamer in each region.

The development of a biofilm (Case 1 in Table 1) over 6 days is shown in Fig. 2a. The top-left image shows the height map *h*(*x*, *y*) of the biofilm after 12 h, followed by a time step of 24 h between consecutive images. During the first day, simple structures with a height *h* of up to 175 μm form in a sparse pattern. Over time, the structures grow, increasing in size and complexity. Starting from the second day, the typical structure becomes wider and may begin developing a streamer, i.e. a thin filament that extends downstream from its tip. These streamers are characterised by a low solidity ratio (*T*/*h*), which quantifies the proportion of biofilm material beneath the bulk-biofilm interface relative to the voids (Fig. 1b). Two regions that contain significant streamer development are shown in detail in Fig. 2b. These regions correspond to the highlighted boxes in Fig. 2a. A vertical slice through these regions can be found in Supplementary Fig. 1.

The biofilm morphology can qualitatively be described as a collection of microcolonies, schematically shown in Fig. 1a. Each microcolony consists of one base—shaped as a leaning pillar—that may have a streamer attached to the tip of the base. These streamers are aligned with the flow, with adjacent structures creating the appearance of continuous lines in the streamwise direction. This is clearly shown in region 1 (left frame in Fig. 2b), where streamers originate from three separate biofilm structures that are connected into one aggregate. During days 4 to 6, the number of microcolonies increases over time, progressively filling the measurement area. No major sloughing events are observed. One time series of height maps from each case is available in Supplementary Figs. 3–8.

#### Streamer formation

Based on recent understanding^8,13,14^, the formation of streamers occurs in two steps. First, aggregation of floating cells is caused by geometry-induced secondary flows. In the second step, a streamer develops as the bioaggregate is extruded by the tangential fluid shear stress. In order to assess whether the same mechanisms underlie the streamers observed in our experiments, we performed numerical simulations and a time-resolved experiment.

**Fig. 3:**
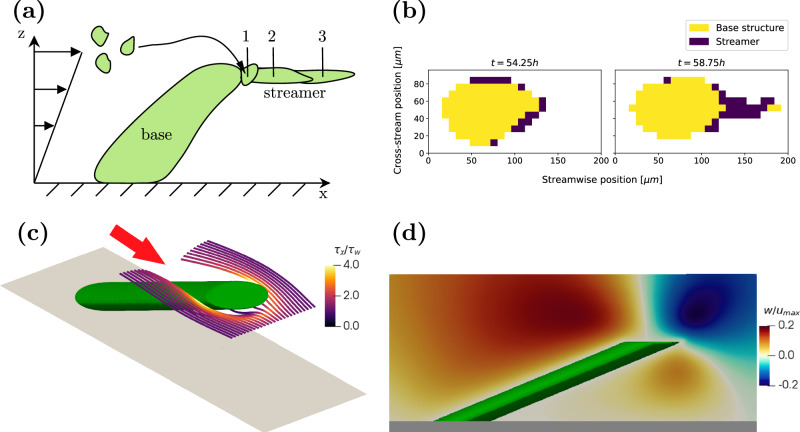
Streamer formation. Frame **a** illustrates the mechanism of streamer formation. Floating particles are transported by the flow until they reach an attachment point (1). They are then stretched by fluid forces (2) and (3) until they reach their final length. Frame **b** shows a microcolony at *t*_1_ = 54.25 h and *t*_2_ = 58.75 h. Between these two instances, a streamer with a length of approximately 50 μm develops. Frames **c** and **d** visualise the flow field around a leaning pillar in a shear-driven flow at *Re*_*cyl*_ = 1, representative of a microcolony from Fig. 2. **c** shows streamlines, while **d** depicts the vertical velocity component in the centre-plane of the cylinder. The flow direction is indicated by the red arrow, with secondary motions converging downstream, near the pillar tip, where longitudinal shear increases to roughly three times the driving shear stress.

As explained earlier, the streamers in our experiments originate from the leeward side near the tip of the base structures. From numerical simulations (details provided in “Numerical simulation”) of the flow around a simplified rigid base structure with the shape of a tilted cylinder, we observe that streamlines originating upstream curve around the cylinder and converge directly downstream of the tip (Fig. 3c). This is associated with a secondary motion at the tip of the cylinder, which is quantified by the vertical velocity component *w* (Fig. 3c). There is a vertical transport of floating cells and nutrients from above (negative *w*) and below (positive *w*), enhancing the capture of floating material at the tip of the cylinder.

Moreover, the longitudinal shear stress immediately downstream of the tip of the cylinder is elevated to three times the wall shear stress, facilitating the stretching of the captured material into a streamer. This extrusion is due to fluidisation, i.e., viscous deformation, and occurs on a much shorter timescale than the biological growth of the base structure. To identify these time scales, we grew a biofilm under the conditions of Case 4 (see Table 1) in a channel and measured the extent of the biofilm every $15\,\min$. Figure 3b shows the development of a single representative colony. The biofilm is separated into a base structure and a streamer using the solidity *T*/*h*. Regions with *T*/*h* ≤ 2/3 are classified as streamers and depicted in blue, while the base structure is shown in yellow. A full representation of the time series is available in Supplementary Fig. 2.

The first frame represents the microcolony 54 h after its initial appearance. While a small region on the perimeter of the colony fulfills the streamer criterion, no filament has yet formed. Between *t* = 55.25 h and *t* = 58.25 h, a filament is intermittently visible. This intermittency is likely caused by the low cell concentration within the streamers, as observed by Savorana et al.^4^, reducing the contrast between the biofilm and surrounding fluid. After *t* = 58.25 h, the streamer becomes dense enough to remain consistently visible. Over this period, a streamer with a length of ~50 μm forms within 3 h, i.e. much faster than the development of the base structure that takes roughly 2 days. We thus observe that given a smooth wall, the biofilm itself can create a geometric defect that then enables streamer formation. This process may explain the results in one recent study, which for the first time observed the formation of streamers on flat surfaces in undisturbed laminar flow^18^.

We may estimate the viscosity of the biofilm by assuming that streamer is fluidised, where the local shear stress *τ*_s_ is proportional to the normal strain rate. If *L*_0_ and *L*(*t*) denote an initial and final length of the streamer, then, the normal strain rate is given by,

1 $$\frac{1}{{L}_{0}}\frac{\Delta L}{\Delta t}=\frac{{\tau }_{s}}{{\mu }_{{\rm{b}}}},$$

where *ΔL* = *L*(*t*) − *L*_0_ and *μ*_*b*_ is characteristic viscosity of the biofilm. From our time-resolved experiments (Fig. 3b), we find *ΔL*/*L*_0_ ≈ 10 and *ΔT* ≈ 1 × 10^4^ s. Assuming that the local shear stress is three times the wall shear stress results in *τ*_*s*_ ≈ 1 Pa, we obtain a biofilm viscosity of *μ*_b_ ≈ 1 × 10^3^ Pa, matching the estimate by Rusconi et al.^8^ and close to the viscosity of a *Bacillus subtilis* grown on an agar plate^3^. This indicates that the streamer does, in fact, expand via a process of fluidisation.

#### Influence of wall shear on biofilm morphology

**Fig. 4:**
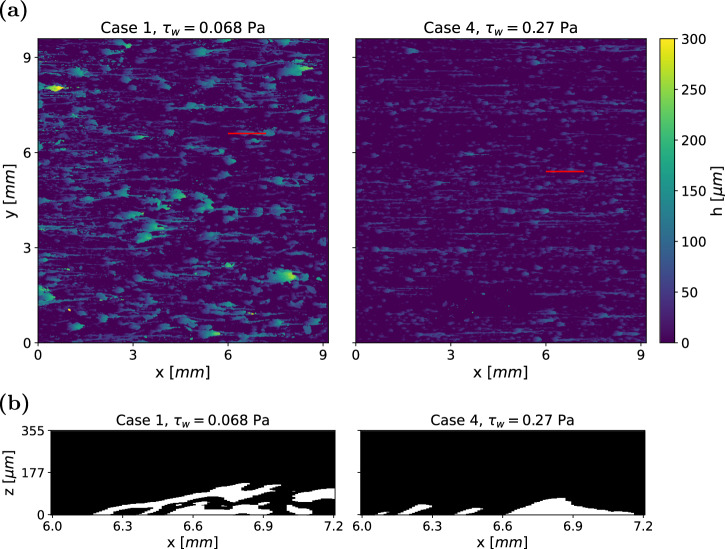
Comparison of two biofilms grown under different hydrodynamic conditions: Case 1 and 4. **a** Height maps of two biofilm samples after 6 days of growth, with the flow direction oriented from left to right. **b** Representative biofilm structures along the red lines in (**a**). Under higher wall shear stress, the biofilm forms more compact structures.

To investigate the influence of different wall shear stress levels on biofilm morphology, we compare the structures of two biofilms grown under varying conditions (Cases 4 and 1 in Table 1). The biofilms, which are six days old, are shown in Fig. 4a. The biofilm subjected to a lower wall shear stress of *τ*_*w*_ = 0.068 Pa developed taller and wider structures compared to the biofilm grown under a higher shear stress of *τ*_*w*_ = 0.27 Pa. Furthermore, the structures (including streamers) in Case 1 are significantly larger than those in Case 4.

A side view of the slices that are marked by red lines in Fig. 4a is shown in Fig. 4b. While Case 4 primarily consist of small, leaning pillars, Case 1 exhibits more complex formations. Large sheets of biofilm extend from small contact points with the substratum. The segment of biofilm in Case 1 that appears detached from the substratum is, in fact, connected to the adjacent biofilm on a neighbouring slice. The formation of more compact biofilms under increased wall shear stress is consistent with previous observations^20,21^.

**Fig. 5:**
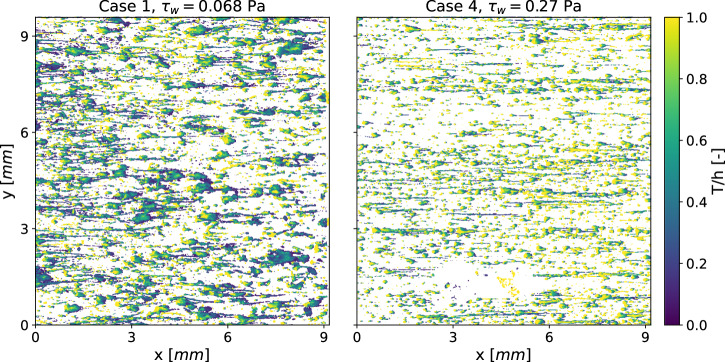
Material distribution of two sample biofilms after 6 days. Low values indicate proportionally large voids within or beneath the biofilm.

The increased compactness of the biofilm is further illustrated by the vertical distribution of biofilm, represented by the solidity ratio *T*/*h*, as shown in Fig. 5. A value of one (yellow) indicates an uninterrupted column of biofilm from the substratum to the liquid-biofilm interface, while values near zero (dark green) signify thin films with voids beneath, such as streamers. The characteristic microcolony structure, consisting of a base with an attached streamer (Fig. 1a, is clearly visible at the higher wall-shear stress (Case 4 in Fig. 5, right frame). Here, bright yellow regions correspond to base structures, while dark green areas represent streamers that are disconnected from the substratum.

In the low shear stress configuration (Case 1 in left frame of Fig. 5), the microcolonies are generally larger but of similar shape. This suggests that while shear stress influences the size of biofilm structures, it does not alter the fundamental streamer formation process. Even small contact patches can support large biofilm structures, whereas higher shear stress limits the size and shape of these structures. The typical microcolony morphology, consisting of tilted pillars with an attached streamer, is consistently observed across all the datasets.

#### Changes in the substratum coverage

**Fig. 6:**
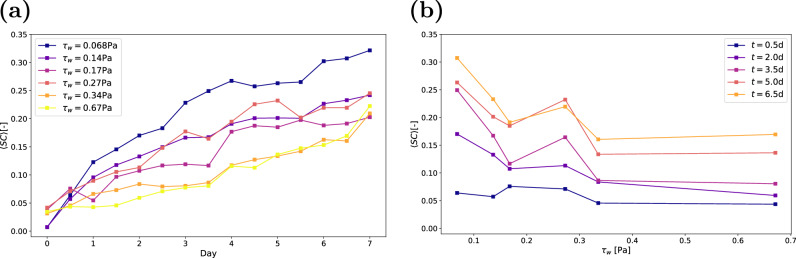
Substratum coverage. **a** Substratum coverage over time, where each point represents the mean value of all replicates. **b** Coverage as a function of wall shear stress, based on the mean value of all replicates. Data is shown for five regularly spaced measurements.

The substratum coverage, *SC*, defined as

2 $$SC(t)=\frac{1}{A}{\int}_{A}b(x,y,0,t)\,{\rm{d}}A,$$

describes the proportion of the substratum that is directly covered by biofilm at time *t*. Figure 6 shows the time evolution of substratum coverage for the six configurations listed in Table 1. The data represent ensemble averages, calculated by averaging *SC* across all replicates. During the first seven days, the substratum coverage increases almost monotonically. While several instances exist, e.g. on day 4 for the biofilms grown under *τ*_*w*_ = 0.068 Pa, where the substratum coverage decreases, these reductions are much smaller than the confidence intervals of the measurements and could not be linked to specific sloughing events. Individual graphs for each condition, including the confidence intervals, are provided in Supplementary Figs. 9–14. Our results are consistent with previous measurements by Gierl et al. who studied the growth of *Bacillus subtilis* at a wall shear stress similar to Case 1^17^. In their study, surface coverage reached even higher values, approaching *SC* ≈ 1. However, Gierl et al. defined *SC* differently, using the maximum intensity projection of *b* along the vertical axis instead of the biofilm directly in contact with the substratum *b*(*x*, *y*, 0, *t*).

It is evident that as wall shear stress increases, substratum coverage decreases, a trend consistent with previous studies^18,23^. The increase in substratum coverage at *τ*_*w*_ = 0.27 Pa is caused by one outlier, where the coverage is 3.5 times larger than the mean of the other measurements. Figure 6a shows *SC* as a function of wall shear stress at different time instances. We observe that the substratum coverage in the first 12 h is nearly constant with the shear stress. However, by day two, cases with lower shear stress exhibit significantly higher *SC*. With coverage that exceeds 10% after 3 days, interactions at the substratum between neighbouring colonies such as observed by ref. ^29^ become much more likely. These interactions may influence the further spreading of either colony, thus complicating the relationship between substratum coverage and time. Additionally, a small number of colonies detach between some measurements, a process likely to become more prevalent as time passes.

Overall, our data demonstrates that the shape and size of the structures as well as the substratum coverage are sensitive to the shear stress. Our work additionally highlights that, as previously shown in turbulent flows^5^, streamers can form on the edges of the biofilm base structures in canonical laminar channel flows. In the following section, we further quantify the relationship between biofilm growth and shear stress and explain our observations with a simple model.

### Analysis of the relationship between biovolume and shear stress

**Fig. 7:**
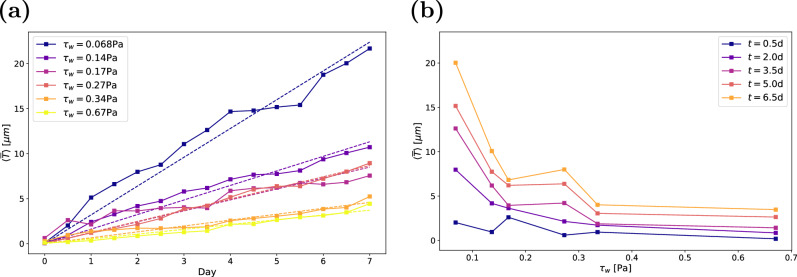
Change in biovolume. **a** Mean biofilm thickness over time, with dashed lines indicating the best fit for $\overline{T}(t)=at$. **b** Mean biofilm thickness as a function of wall shear stress. Each point represents the average of three or four samples in both **a** and **b**.

The accumulation of biofilm can be quantified by the volume of the biofilm *V*_*b*_(*t*) = ∫_*V*_*b*(*x*, *y*, *z*, *t*) d*V*. Here, we use the mean biofilm thickness $\overline{T}(t)={V}_{b}(t)/A$ as a normalised measure of the biovolume. This accounts for differences in the field of view that result from reflections and noisy regions within the measurements. Figure 7a shows the development of mean biofilm thickness with time, averaged over all replicates at the same condition. We find a continuous growth of the biofilm and that the accumulation of biofilm strongly depends on the wall shear stress. With increasing wall shear stress, the growth rate decreases. Individual graphs for each condition, including the confidence intervals, are provided in Supplementary Figs. 15–20. Despite a scatter between the individual measurements at the same shear stress, the averaged data reveal a clear trend: mean biofilm thickness grows approximately linearly in time. Similar growth behaviour in time has been reported previously^15,16^. Gierl et al.^17^ also observed nearly linear accumulation of *Bacillus subtilis* NCIB 3610 biofilm at a wall shear stress close to *τ*_*w*_ = 0.068 Pa, which is the lowest wall shear stress under consideration here.

Figure 7b shows the mean biofilm thickness as a function of the wall shear stress. Here, a clear trend of decreasing biovolume with increasing wall shear stress is visible at all times. The increase in mean biofilm thickness at *τ*_*w*_ = 0.27 Pa (Case 4) again stems from an increased biofilm growth in one channel. The decay of the biovolume with the wall shear stress confirms the often-observed trend that increasing wall shear stress impedes biofilm development^18,30,31^. We performed a nonlinear least-squares fit of the form $\overline{T}(t,{\tau }_{w})=at{{\tau }_{w}}^{k}$. The resulting coefficients (±standard deviation) are *a* = 0.29 ± 0.04 and *k* = −0.89 ± 0.06, revealing an approximately inversely proportional relation between the mean biofilm thickness and the wall shear stress.

#### Mechanisms of friction-limited growth

From the observed trends in Fig. 7, we may assume that biofilm grows linearly in time and is inversely proportional to the wall shear stress. We additionally observe (Fig. 2a) that the individual microcolonies mature, i.e. reach their final height, within a few days. Thereafter, the volume of each microcolony remains almost constant. Next, we illustrate that these trends can be explained by a balance between erosion and growth.

**Fig. 8:**
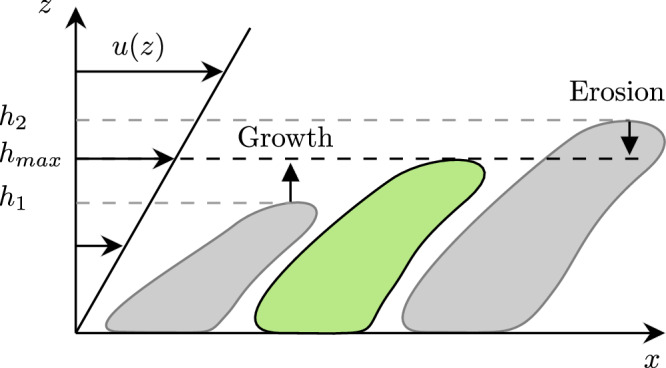
Three microcolonies are exposed to a shear flow. The small colony is growing, while the larger colony is dominated by erosion. The green colony is in equilibrium.

As a simplification, we assume that a microcolony has a constant cross-sectional area. The volume of a microcolony can thus be approximated by *V*_*B*_ ≈ *A*_*fp*_*h*_*mc*_, where *A*_*fp*_ is the footprint area and *h*_*mc*_ is the height of the microcolony. A simple balance equation for *h*_*mc*_ reads,

3 $$\frac{{\rm{d}}{h}_{mc}}{{\rm{d}}t}=g-e,$$

where *g* is the change in height due to growth, and *e* is the height loss rate due to erosion. If *g* > *e*, the microcolony grows, and if *g* < *e* erosion will reduce the height of the colony. This is visualized in Fig. 8. When the right-hand side is zero (*g* = *e*), the microcolony has reached an equilibrium height ${h}_{\max }$ (green-centred structure in 8). We model *g* as a constant with a value determined by the biological properties of the biofilm. In general, the growth rate can be modelled with a Monod term to account for the effects of nutrient gradients. However, here we want to focus on friction-limited growth and thus assume that the flow is fully mixed and saturated with nutrients and oxygen.

For simplicity, we assume that erosion the *e* is linearly proportional to the imposed fluid shear stress and the height of the microcolony, i.e., *e* ∝ *h*_*mc*_*τ*_*w*_. The dependence on *h*_*mc*_ reflects the greater local viscous forces experienced by taller microcolonies. Indeed, with an increasing height, the flow velocity at the microcolony tip increases, and hence does the local viscous force, which effectively erodes the colony. We may write

4 $$e=\frac{C}{{\mu }_{b}}{h}_{mc}{\tau }_{w}$$

where *μ*_*b*_ is a reference effective viscosity of the biofilm. Our earlier estimate of the biofilm viscosity (based on shear–stress-induced expansion of streamers using equation (1)) gave *μ*_*b*_ ≈ 1 × 10^3^ Pa. In Eq. (4), *C* is a dimensionless coefficient that incorporates biological, mechanical and other effects that may influence biofilm erosion. We assume that *C* is independent of time and shear stress. Prior studies have indicated that the biofilm viscosity depends on the wall shear stress that is imposed during its growth^2,6^. However, to best of our knowledge, there is no quantitative data on this relationship. Note, that this is different from rheological investigations, where the viscous response of a fully developed biofilm is quantified. In this study, we assume a weak or negligible dependence of *μ*_*b*_ on imposed shear stress.

By inserting (4) into the balance Eq. (3) and assuming an equilibrium state (d*h*_*mc*_/d*t* = 0), we obtain the maximum height that a microcolony can reach:

5 $${h}_{max}=\frac{g{\mu }_{b}}{C}\frac{1}{{\tau }_{w}}.$$

We thus note that a height (or equivalently mass) balance combined with a linear constitutive relation for the erosion results in an inverse relationship between the equilibrium height of the microcolony and the imposed shear stress. The local fluid velocity at a height *h*_*max*_ is given by *u*_*max*_. Near the wall, this velocity is

6 $${u}_{max}({\tau }_{w})={h}_{max}\dot{\gamma }=\frac{g{\mu }_{b}}{C}\frac{1}{{\tau }_{w}}\frac{{\tau }_{w}}{\mu }=\frac{g}{C}\frac{{\mu }_{b}}{\mu }.$$

Thus, a microcolony will grow until it is exposed to a flow with a characteristic velocity that is determined by the biological growth rate and the ratio of the viscosities of the biofilm and medium.

As described in “Biofilm morphology”, the measured biofilms consist of many microcolonies. Here, we assume that these colonies are independent of each other and appear at a constant rate *β*, such that the number of microcolonies *N*_*mc*_ is given by

7 $${N}_{mc}=\beta t.$$

This is supported by the approximately linear growth of the substratum coverage in Fig. 6b, which corresponds to SC = *N*_*mc*_*A*_*fp*_/*A* with *A*_*fp*_ as the footprint area of a microcolony and *A* as the total measurement area. Since the individual microcolonies mature quickly, we assume all colonies to be in their equilibrium state. If each microcolony additionally has a constant and equal footprint *A*_*fp*_, the collective biofilm volume of the microcolonies is given by

8 $${V}_{b}={N}_{mc}{A}_{fp}{h}_{max}.$$

Combining Eqs. (5) and (7), we get

9 $$V_b = \underbrace{\left(\beta A_{fp} \frac{g\mu_b}{C}\right)}_{=K} \frac{t}{\tau_w}.$$

The term *K* encapsulates biological, material and geometrical features of the biofilms and needs to be determined empirically, but is independent of time and wall shear stress. The biofilm volume can be normalised on a region of interest, or field of view, *A*, to obtain the final expression in our model

10 $$\overline{T}=\frac{K}{A}\frac{t}{{\tau }_{w}}.$$

This qualitative model explains the behaviour that we observe in Fig. 7, namely linear growth in time, and inverse scaling with the wall shear stress.

## Discussion

We have conducted a study on the influence of wall-shear stress on the growth of *Bacillus subtilis* biofilm in rectangular channels at low Reynolds numbers. Due to the wide-aspect ratio of the channels, it can be assumed that the external flow is a plane Poiseuille flow. Combining a model organism and canonical laminar flow enables a fundamental study of biofilm growth. We have used an experimental setup where biofilms are grown in 12 channels simultaneously at different shear stresses. In this way, we have reduced variations in growth that may be caused by disturbances in the external environment. Using optical coherence tomography, we obtained full volumetric scans of the biofilms every 12 h over 6 days without disturbing the samples.

We found that biofilms can be regarded as a collection of microcolonies where each colony has a base structure in the form of a leaning pillar and a streamer in the form of a thin filament. While the shape, size, and distribution of these microcolonies depend on the imposed shear stress, the same structural features were observed for all shear stress values. To the best of our knowledge, this study is the first to report the consistent and robust formation of these microcolonies in laminar flow across an order-of-magnitude interval in shear stress. The time evolution of the biofilms suggests that the base of the microcolony develops first within the first 1–2 days. Once the base is formed, secondary motions occur around the pillar and converge on its leeward side. Previous work^8^ has demonstrated that secondary motions near geometrical disturbances trigger the formation of streamers. In our case, streamers form on the leeward side close to the tip of the microcolonies, suggesting that this mechanism can be triggered not only by the geometry of the channel but also by the shape of the developing biofilm. To observe the evolution of such microcolonies, the time window needs to be longer than hours to allow for the sequential forming of the base structure followed by a streamer but shorter than months to avoid a more complex, fully connected biofilm morphology. Indeed, we observe that with time, the microcolonies merge either through streamer growth or through the expansion of the pillars. Interestingly, the alignment of streamers of the many microcolonies results in a distinct macroscopic morphology characterised by a pattern of long, narrow, and elongated streaks.

Our second main contribution is more quantitative and related to how the biofilm volume depends on shear stress over time. We observed that the amount of biofilm within a channel, measured by its volume, grows approximately linearly over seven days for all the shear stress values. Furthermore, the growth rate was inversely proportional to the wall shear stress. This behaviour can be explained with a simple model based on formulating a mass balance of the biofilm. We assumed an equilibrium state such that the rate of erosion is balanced by the growth of the biofilm. Further, assuming a linear relationship between erosion and shear stress, we could show that the biofilm volume at equilibrium is inversely proportional to the external shear stress. A second insight obtained by observing the biofilm growth was that the number of microcolonies increased over time. Using this fact, we could arrive at a final expression for the biofilm volume that scales with time and shear stress in agreement with our observations.

The model provides valuable insight into a friction-limited growth mechanism. Having assumed that we do not have nutrient depletion, a surprisingly simple relationship seems to exist between fluid forces and biofilm growth of *Bacillus subtilis* for intermediate times. Continuum models where the biofilm is modelled as an active matter may thus explicitly incorporate the dependence of shear stress and decouple the friction-limited growth rate from the nutrient-limited one. Another implication is that time and shear stress are decoupled in expression (9). This means that two biofilms grown under different shear stresses reach the same stage at different times. For example, reducing the shear stress by a factor of two results in the same biofilm volume as the original shear stress after twice the growth time.

Our conclusions are based on one specific bacterial species assuming well-mixed conditions and for intermediate times. The observed growth mechanism is only applicable as long as new, independent structures appear. Future work will explore whether the specific form of micro-colonies and their growth mechanisms observed here also holds for other species and flow conditions.

## Methods

### Flow conditions

The wall shear stress in a wide channel (without biofilm) can be estimated from plane Poiseuille flow as

11 $${\tau }_{w}={\left.\mu \frac{\partial u}{\partial z}\right| }_{z = 0}=\mu \frac{6}{H}{U}_{b}=\mu \frac{6Q}{{H}^{2}w},$$

where *μ* is the dynamic viscosity, *U*_*b*_ the bulk velocity, *Q* the flow rate, *H* the channel height, and *w* the channel width. In our experiments, the average height of the biofilm remains below 3% of the channel height. Therefore, the estimated wall-shear stress is not adjusted over time.

The wall shear stress (11) can be varied in two ways: by changing the flow rate in a given channel^30^, or by a change in channel height at a given flow rate^18,19^. Here, the biofilms are grown in channels of three heights ($1\,\,\text{mm}\,,\sqrt{2}\,\,\text{mm}\,,2\,\,\text{mm}$) and the experiments are conducted at two fixed flow rates: 1 mL s^−1^ and 2.63 mL s^−1^.

**Fig. 9:**
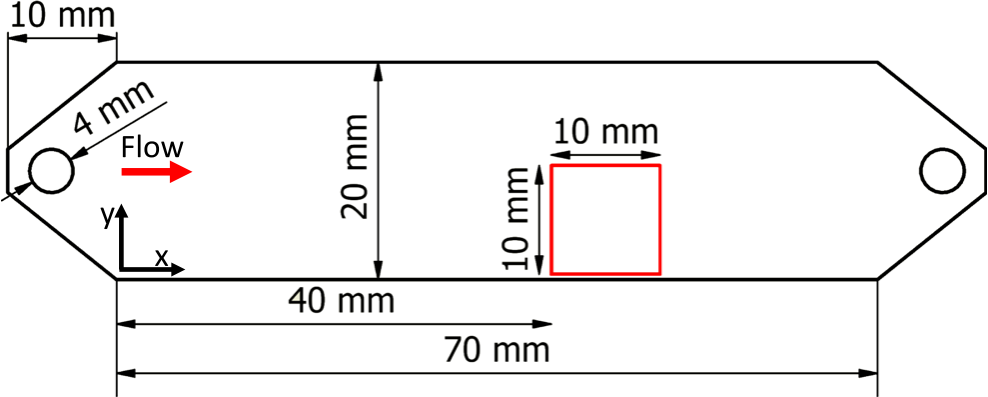
Channel geometry. The measurement area (FOV = 1 cm^2^) is highlighted in red.

Figure 9 shows a top view of the geometry of one channel. All channels have a width of 20 mm and a length of 70 mm with an aspect ratio (width-to-height) of at least *AR* = 10, minimising the influence of the side walls. To avoid the formation of jets and thus reduce the entrance length, the medium is introduced to the channel in the vertical direction. The channels are made of milled polyoxymethylene (POM). Optical access is provided through the top of the channel, which is made of polymethyl methacrylate (PMMA). Due to the different loads on the pumps, the temperature inside the system stabilised at 24 ^∘^C and 27 ^∘^C, respectively. This elevated temperature at the higher flow rate contributed to a slight increase in the growth rate *g*^32^. However, this effect was deemed minor, as we observed a consistent trend across all samples (see Fig. 7b). Additionally, the temperature difference led to a change in the viscosity of the water, which was accounted for in the calculation of flow properties. The experiments were conducted at Reynolds numbers of Re = *U*_*b*_*D*_*H*_*ρ*/*μ* = 100 and Re = 300. Here, *D*_*H*_ is the hydraulic diameter and *ρ* is the density of water. The relevant parameters for the six configurations are listed in Table 1.

**Fig. 10:**
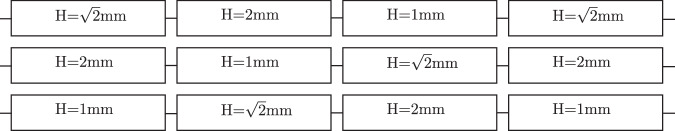
Ordering of channels. The channels are ordered such that each channel height is placed in each streamwise position once.

**Fig. 11:**
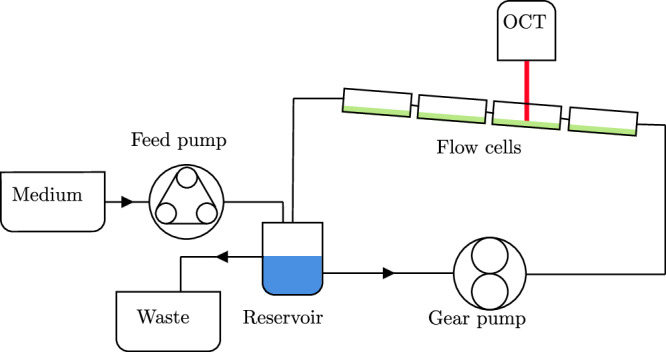
Setup of one flow loop. Three independent flow loops are used.

Twelve channels are set up in three independent flow loops, where each loop consists of four channels in series. The channels are ordered such that each channel height is placed once in each streamwise position. The position of the channels within the larger setup is visualised in Fig. 10. This results in three biological replicates with one additional technical replicate in each condition. One flow loop is shown in detail in Fig. 11. Each flow loop is driven by a magnetic gear pump (Niemzik PAT, Haan, Germany). The cultivation medium is recirculated and fresh medium is extracted from a 10 L bottle by a peristaltic pump and added to the reservoir. The medium in the reservoir is constantly aerated to ensure sufficient oxygen supply. Mixing is provided by magnetic stirrers. The volume in the reservoirs is kept constant by an outlet at the target height. The cumulative residence time of the medium in the flow cells before it is returned to the well-mixed reservoir is below 15 s. Given these conditions, we can assume a steady state in the bulk liquid without nutrient depletion causing a concentration gradient in the streamwise direction.

During the experiment, the flow rate in each flow loop is monitored periodically. Once the flow rate drops sharply, indicating rapid blockage somewhere in the flow loop, the measurement is stopped. This occurred on the eighth day. Additionally, measurements are inspected for artefacts, such as bubbles or large aggregates of biofilm getting stuck in the flow cell. Scans including these are removed from the evaluation.

### Culture preparation

*Bacillus subtilis* NCIB 3610 is taken from frozen stock, plated, and a single colony is resuspended in liquid LB (Lennox) medium. The liquid culture is incubated overnight at 30 ^∘^C with shaking at 140 rpm and diluted to an optical density of OD_600_ = 0.1. The working liquid in the channel is a volumetric 1:100 solution of LB (Lennox) broth in tap water (for mineral content, see ref. ^33^, data for July 2023). The chemical oxygen demand (COD) of the diluted medium is ~250 mg L^−1^. The inoculum is introduced to the flow cells at the target flow rate *Q*. After 1 h, medium replacement is started at a flow rate of *Q*_*r*_ = 1 mg L^−1^ corresponding to a replacement rate of five times per day. The temperature in the medium reservoir is monitored periodically.

### OCT measurements

OCT is an interferometric measurement method that has proven useful for examining biofilm and does not require staining of the sample. OCT is capable of penetrating biofilm much deeper than typical fluorescent microscopy methods, allowing the detection of voids within or beneath the biofilm. Measurements can be taken in situ, without altering the sample. This enables continued observation of the development of a biofilm over an extended period of time. With a voxel size of several microns along each dimension, and a maximum field of view on the order of 1 cm^2^, OCT enables measurements of mesoscopic biofilm structures that are too large for confocal laser scanning microscopy.

The biofilm structure is scanned without interruption of the flow, i.e. without deforming the biofilm due to changing conditions. Measurements are acquired using an OCT system (Thorlabs Ganymede GAN610-SP5) with a central wavelength 930 nm and an LSM04 objective lens. The OCT head is moved to predetermined measurement positions, 45 mm downstream from the inlet, by the automated traverse system introduced by Gierl et al.^17^. The traverse system is an evoBot with a positional accuracy of 0.1 mm^34^. Measurements are taken with a field of view of 10 mm × 10 mm (streamwise × cross-stream) and a lateral pixel size of 12 μm. The sample rate of the OCT is set to 100 kHz. The axial pixel size of the OCT in water is 2.1 μm. A-scan averaging is set to two. Flat interfaces perpendicular to the OCT beam, such as the air-PMMA interface and the PMMA-medium interface, can cause strong reflections and autocorrelation noise in the OCT signal. Therefore, the channels are mounted on a tilted base plate. The angle is set to 5^∘^. The first measurements are taken 2 h after inoculation and then every 12 h. The twelve channels are scanned sequentially within 1 h.

### Data processing and thresholding algorithm

Many different thresholding methods, often Otsu’s method^35^, are used to binarise the OCT scans^36^. These methods struggle to separate the biofilm from the bulk if no bimodal distribution exists in the intensity histogram. Here, we introduce a new thresholding algorithm based on the characteristics of the histogram of a typical OCT scan containing a small amount of biofilm.

**Fig. 12:**
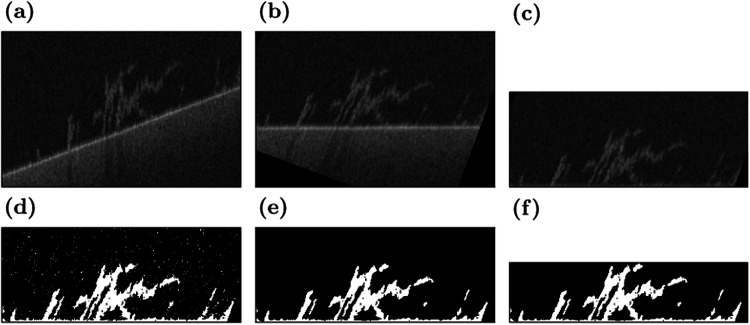
Processing of a sample scan. The raw scan (**a**) is rotated (**b**) and the substratum is aligned with the bottom of the scan (**c**). Then, the image is binarised (**d**), denoised (**e**), and trimmed to the extent of the biofilm (**f**). These images are not to scale to improve visibility.

The OCT imaging software ThorImage returns files in .oct format. This format is an archive that contains metadata and the raw image files in 32-bit floating point greyscale. The raw images are extracted automatically and prepared for processing in ImageJ^37^. The processing steps are visualised in Fig. 12. Since the channels are placed at an angle, the image stacks have to be rotated for the substratum to be oriented horizontally (Fig. 12a). ImageJ is chosen for this step because of its speed at rotating stacks of images. First, the images are converted to 8-bit greyscale, then they are rotated by preset angles along two axes (Fig. 12b). The resulting image stacks are saved.

Further processing is performed using custom Python scripts, which are available at https://www.bagherigroup.com/research/open-source-codes/. First, the substratum is detected by finding the point of highest intensity along the vertical axis and then applying a generous median filter with a radius of eleven pixels. The optomechanical setup of the OCT system causes minor warping in the planar substratum that cannot be removed by the predefined calibration function. Here, this remaining warping is removed by aligning the detected substratum at the bottom of the scan (Fig. 12c).

We developed a thresholding algorithm for our OCT data since existing schemes such as Otsu’s method struggle to accurately detect the biofilm. OCT scans of empty volumes are noisy, with the signal following a Gaussian distribution. The signal that represents biofilm has an intensity that is larger than the mode of the intensity distribution. When the amount of biofilm within a scan is too small to create a bimodal distribution, there is an inflexion point marking the beginning of the biofilm data. This fact is used in the following algorithm. First, the mode of the distribution is detected. Then, the maximum of the second derivative of the intensity distribution to the right of the mode is marked as a candidate threshold. A further offset of three intensity levels is required to reliably match the results of manual thresholding. The resulting threshold is used to binarise the image stack (Fig. 12d). Afterwards, salt-and-pepper noise is removed using a filter similar to the *Remove Outliers* function in ImageJ (Fig. 12e). This filter acts as a median filter removing small biofilm structures, without filling in any gaps. Finally, the image is trimmed from the top to the section containing biofilm to minimise file sizes (Fig. 12f). Once all scans have been processed, consecutive scans of the same flow cell are aligned using cross-correlation-based image registration.

### Numerical simulation

The numerical simulation is performed using BASILISK^38^, an open-source code for the solution of partial differential equations on Cartesian meshes. The flow field is solved using a direct numerical simulation (DNS), solving the incompressible Navier-Stokes equations in a centred formulation. An approximated projection method staggered in time on a Cartesian grid is used, with the prediction of the advection term performed with the Bell-Collela-Glaz second-order unsplit upwind scheme^39^. For the discretisation of the viscous diffusion term, a second-order Crank-Nicholson fully-implicit scheme was used. Spatial discretisation was achieved using an octree grid and the solid wall was described using an immersed boundary (cut cell) method. The numerical simulations were performed at Re = 1.17 based on the biofilm diameter *D* and the velocity at a height corresponding to the tip of the colony *u*_*max*_. The domain size adopted was a square box of size (24*D*, 24*D*, 24*D*), with a mesh resolution varying in size from 0.75*D* in the far-field to ≈0.02*D* on the solid body. Periodic boundary conditions were used in streamwise and spanwise direction, no-slip on the bottom wall and a constant shear, which drives the flow to ensure the prescribed tip velocity, on the top one. The biofilm is placed on the bottom wall, to account for the interaction with the solid surface, at 6*D* from the inlet and in the centre of the domain in spanwise direction to simulate an isolated body despite the periodic boundary condition.

## Supplementary information

The role of fluid friction in streamer formation and biofilm growth—Supplementary information

Biofilm growth over 7 days

## Data Availability

The data that support the findings of this study are available from the corresponding author, C.W., upon reasonable request.
