# Supplementary material for: The role of fluid friction in streamer formation and biofilm growth
Source: NPJ Biofilms Microbiomes. 2025 Jan 15;11:17. doi: 10.1038/s41522-024-00633-2 (PMC11735801; doi:10.1038/s41522-024-00633-2)
Supplement: Supplementary file 1 — The role of fluid friction in streamer formation and biofilm growth—Supplementary information [file 41522_2024_633_MOESM1_ESM.pdf]

The role of fluid friction in streamer formation and biofilm growth – Supplementary information

Cornelius Wittig[1], Michael Wagner[2], Romain Vallon[1], Thomas Crouzier[3], Wouter van der Wijngaart[4], Harald Horn[5], and Shervin Bagheri[1]

[1]FLOW, Dept. of Engineering Mechanics, KTH, Stockholm SE-100 44, Sweden
[2]Karlsruhe Institute of Technology, Institute of Biological Interfaces (IBG-1), Eggenstein-Leopoldshafen, Germany
[3]DTU, Dept. of Health Technology, DK-2800 Kongens Lyngby, Denmark
[4]Division of Micro and Nanosystems, Dept. of Intelligent Systems, KTH, Stockholm SE-100 44, Sweden
[5]Karlsruhe Institute of Technology, Water Chemistry and Water Technology, Engler-Bunte-Institut, Karlsruhe, Germany

1. Animation of biofilm growth

Supplementary Video 1. Animation of biofilm growth of *Bacillus subtilis* over seven days (156 hours) for in a channel flow ($Re = 100 - 300$). The video shows consequentially shear stress values from 0.07 Pa to 0.7 Pa. Each frame in the animations are biofilm height.

CONTACT C. Wittig. Email: wittig@kth.se

2. Vertical slice through streamers

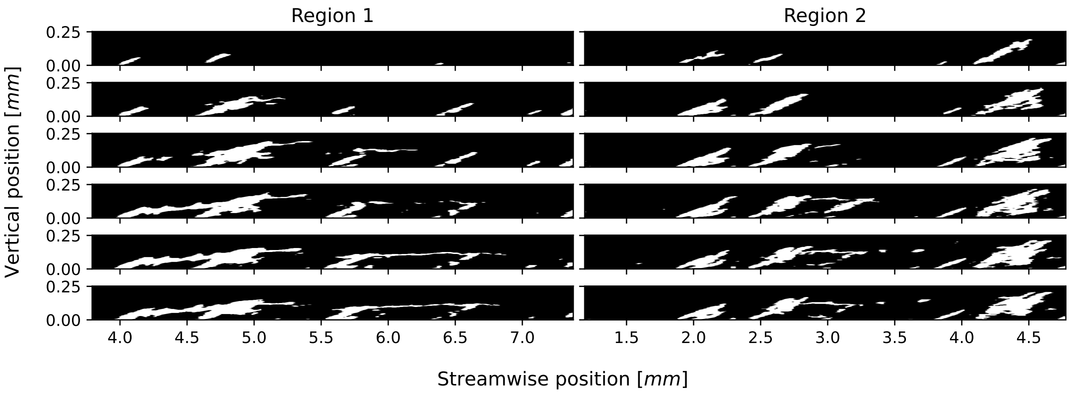

Supplementary Figure 1: **Vertical slices of the streamers in Figure 2b.** The streamers are visible as thin horizontal regions of biofilm, originating on the leeward side of a base, that are suspended above large voids.

3. Evolution of a single streamer

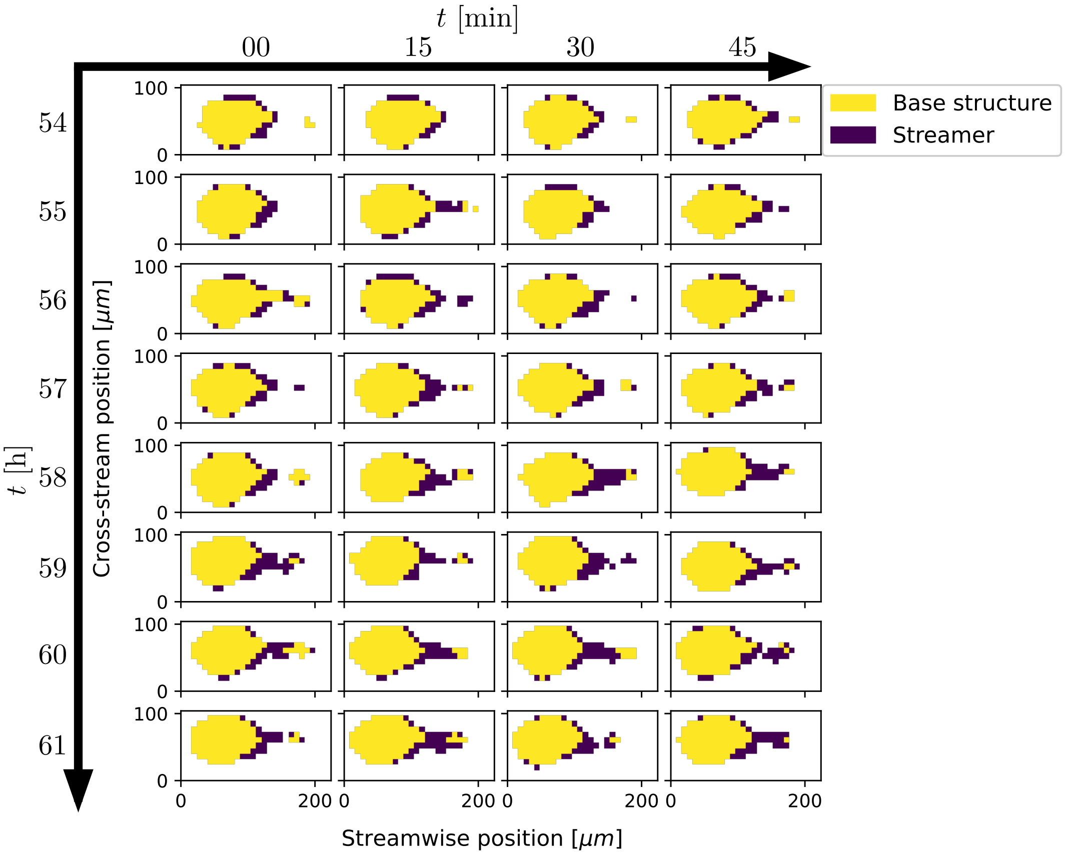

Supplementary Figure 2. **Development of a single microcolony.** The colony is divided into a base structure (yellow) and a streamer (blue). While the base structure develops over several days, the streamer forms within a few hours.

4. Time series data

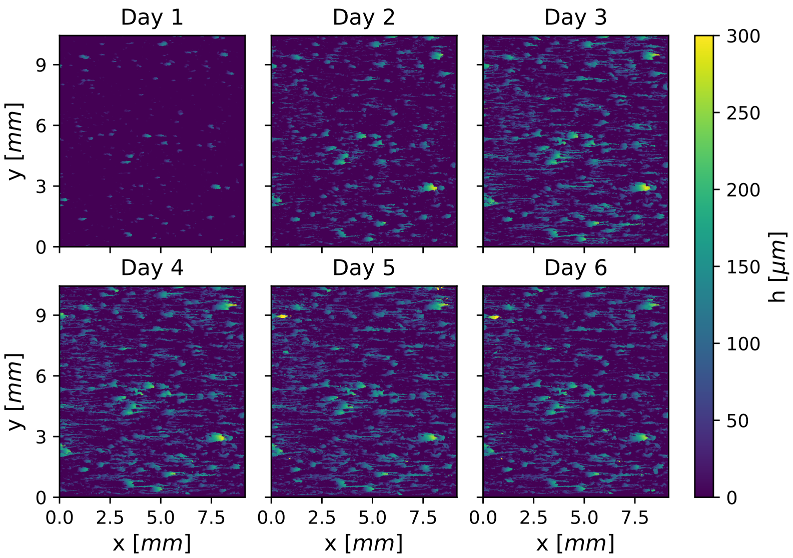

Supplementary Figure 3. Case 1, $\tau_w = 0.068\,\mathrm{Pa}$

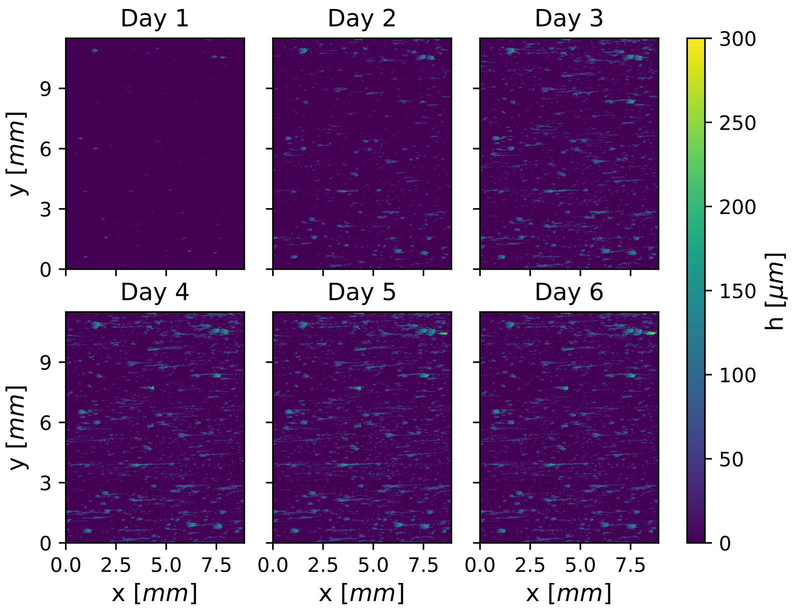

Supplementary Figure 4. Case 2, $\tau_w = 0.14\,\mathrm{Pa}$

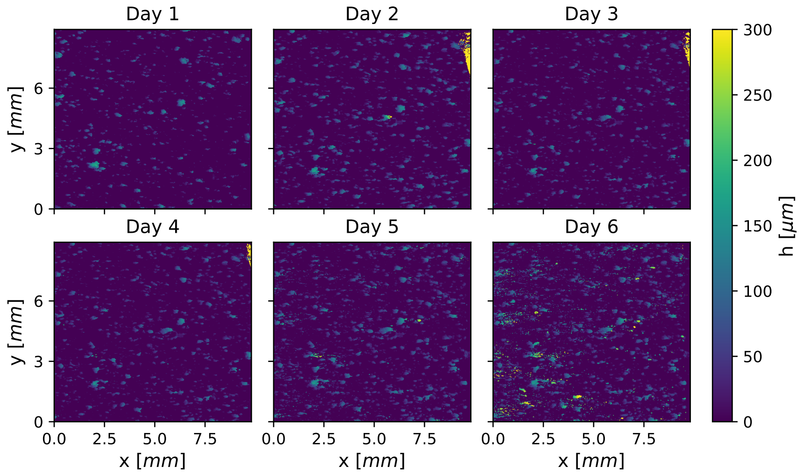

Supplementary Figure 5. Case 3, $\tau_w = 0.17\,\mathrm{Pa}$

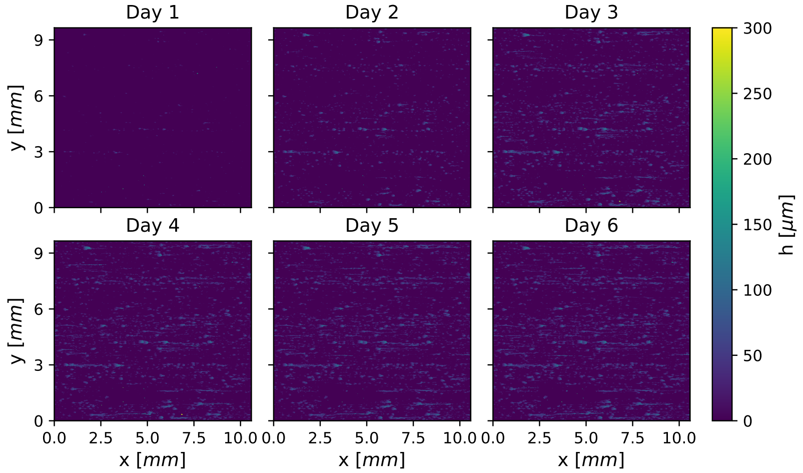

Supplementary Figure 6. Case 4, $\tau_w = 0.27\,\mathrm{Pa}$

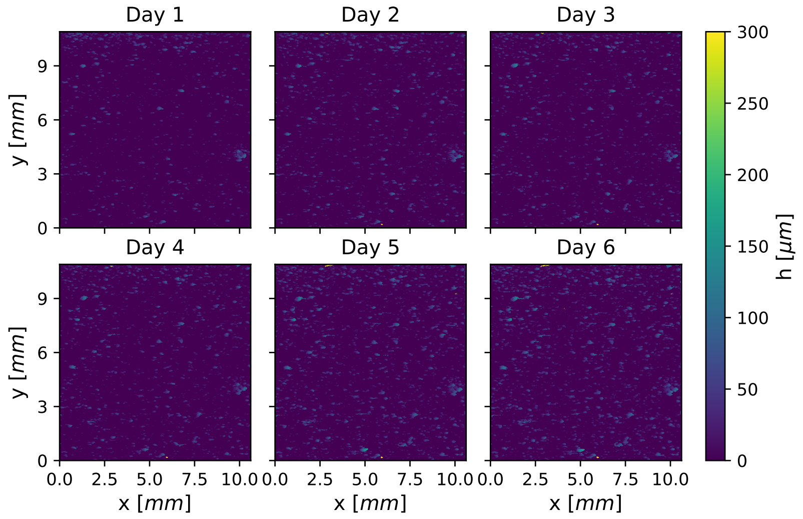

Supplementary Figure 7. Case 5, $\tau_w = 0.34\,\mathrm{Pa}$

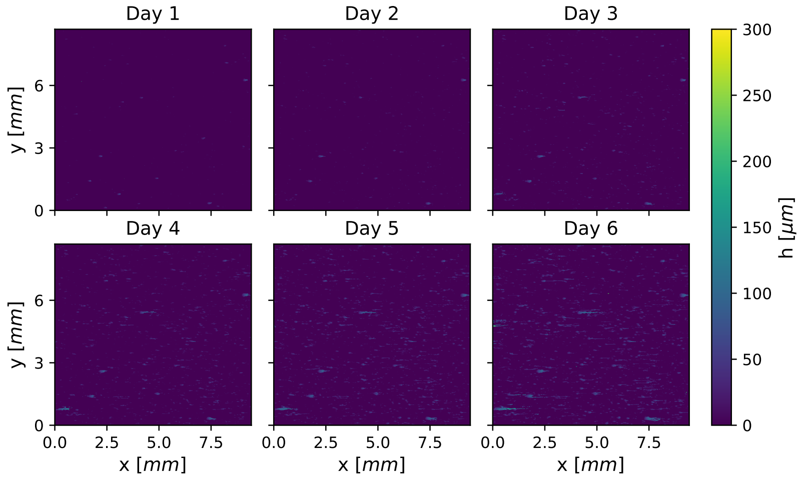

Supplementary Figure 8. Case 6, $\tau_w = 0.67\,\mathrm{Pa}$

5. Confidence intervals of substratum coverage

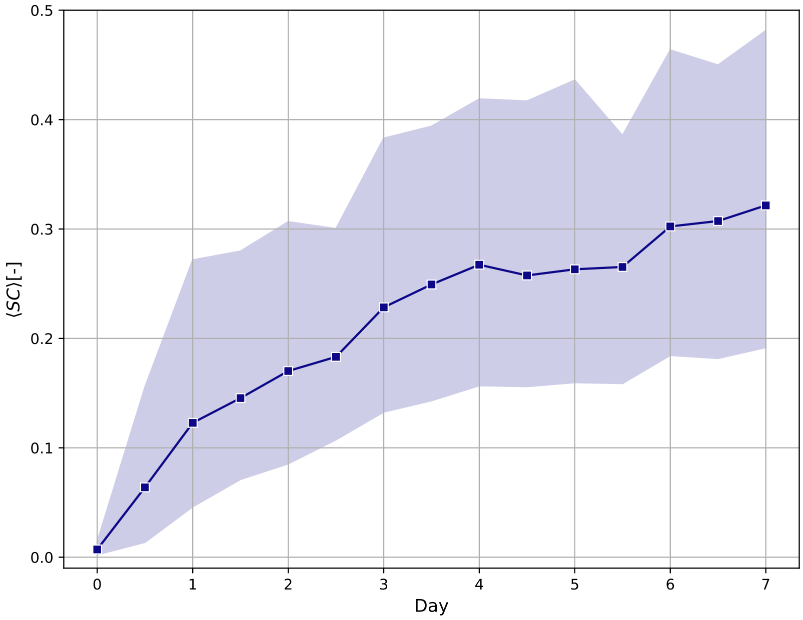

Supplementary Figure 9. Case 1, $\tau_w = 0.068\,\mathrm{Pa}$

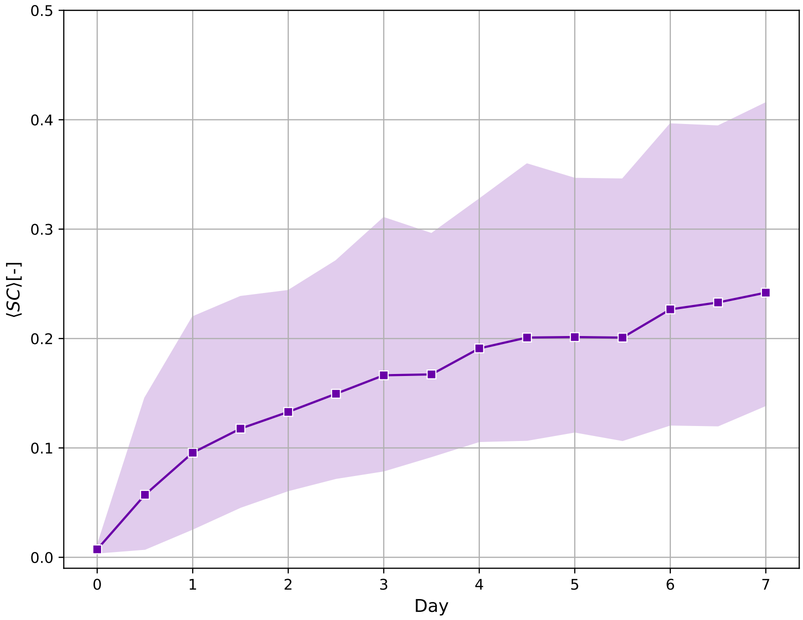

Supplementary Figure 10. Case 2, $\tau_w = 0.14\,\mathrm{Pa}$

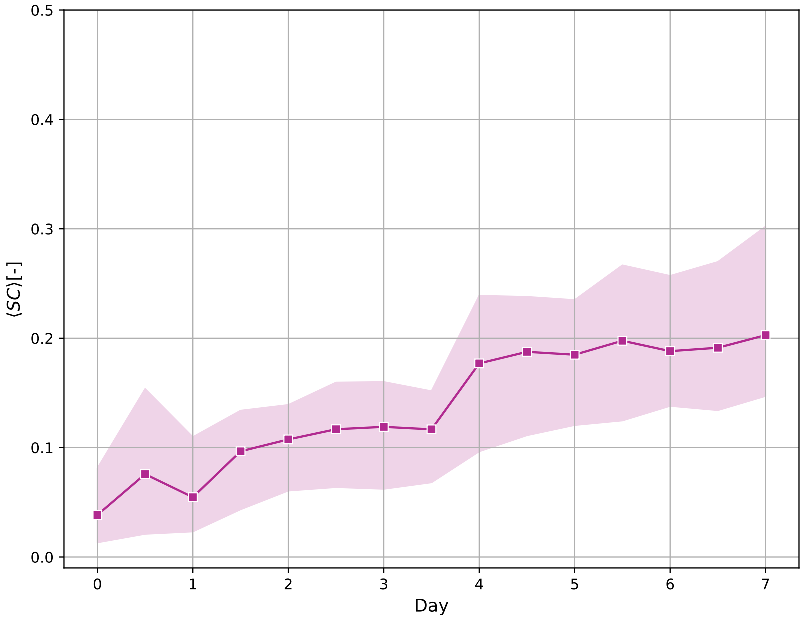

Supplementary Figure 11. Case 3, $\tau_w = 0.17\,\mathrm{Pa}$

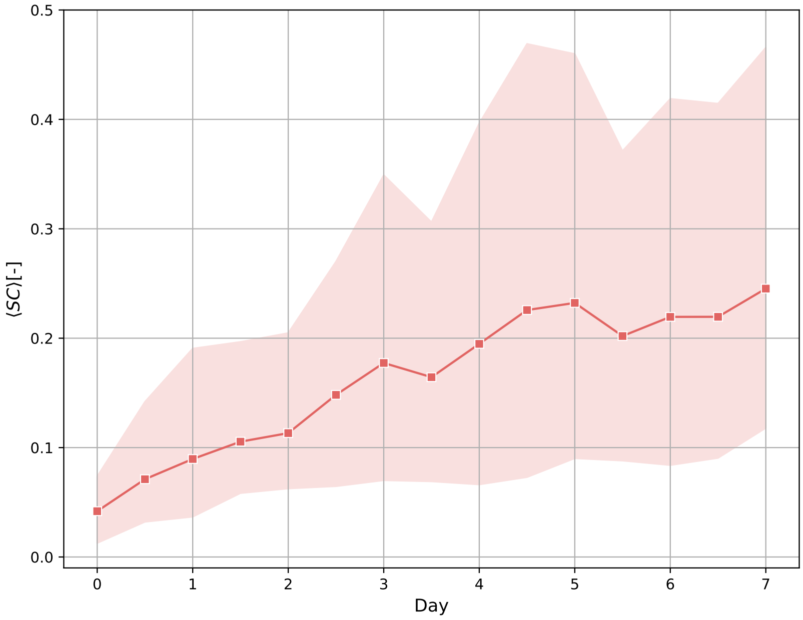

Supplementary Figure 12. Case 4, $\tau_w = 0.27\,\mathrm{Pa}$

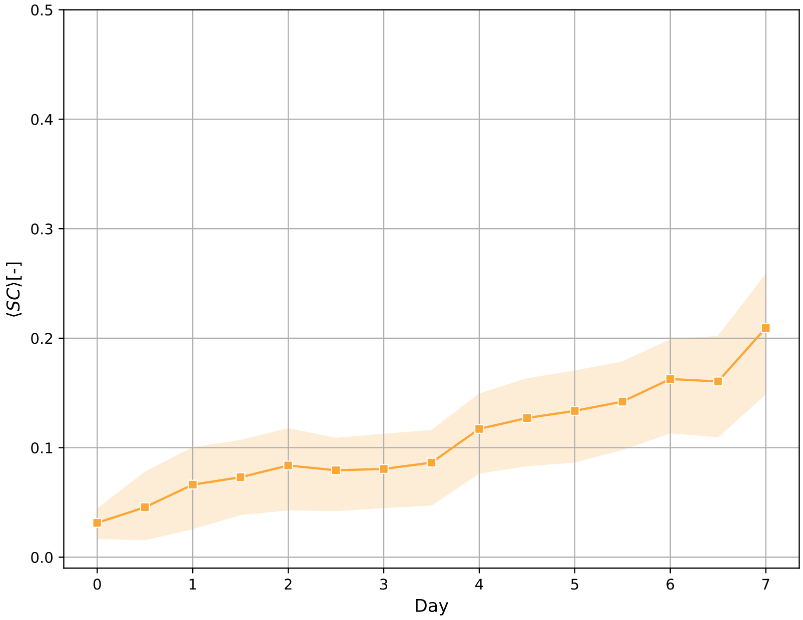

Supplementary Figure 13. Case 5, $\tau_w = 0.34\,\mathrm{Pa}$

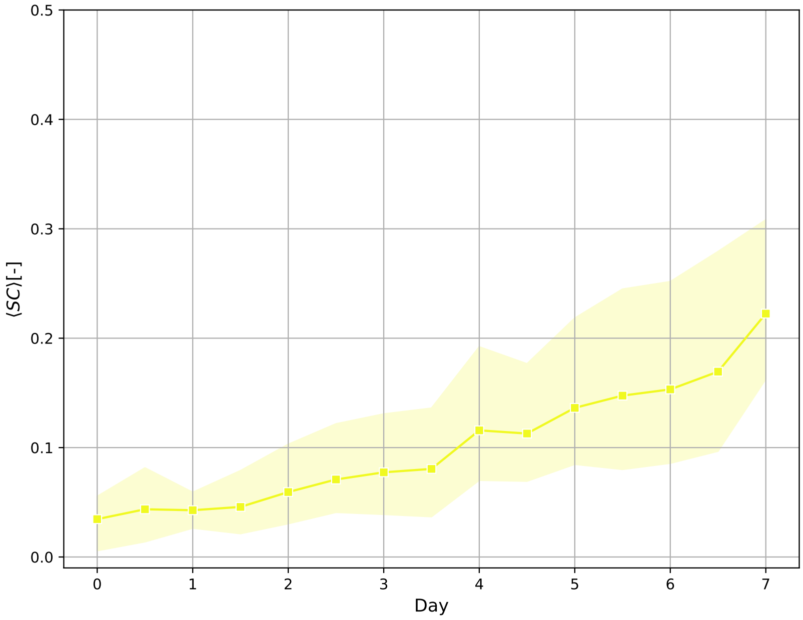

Supplementary Figure 14. Case 6, $\tau_w = 0.67\,\mathrm{Pa}$

6. Confidence intervals of mean biofilm thickness

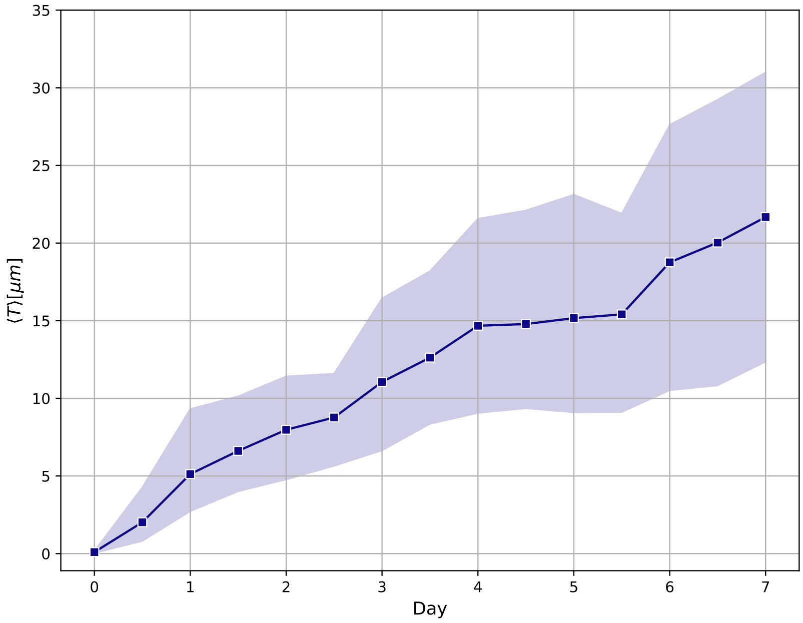

Supplementary Figure 15. Case 1, $\tau_w = 0.068\,\mathrm{Pa}$

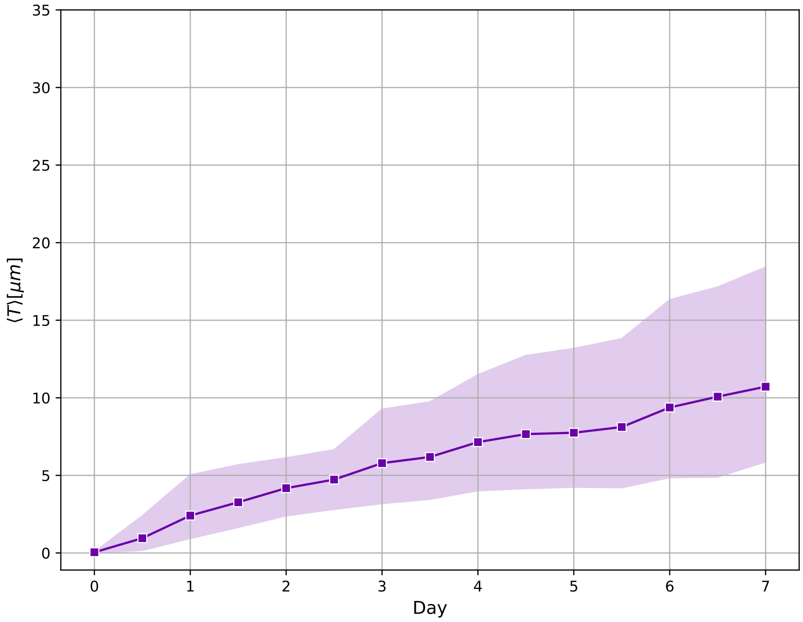

Supplementary Figure 16. Case 2, $\tau_w = 0.14\,\mathrm{Pa}$

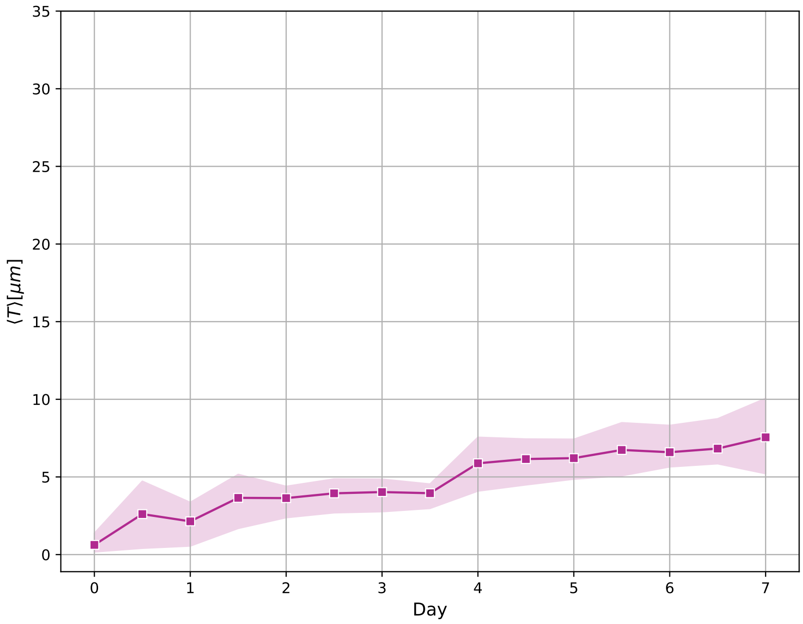

Supplementary Figure 17. Case 3, $\tau_w = 0.17\,\mathrm{Pa}$

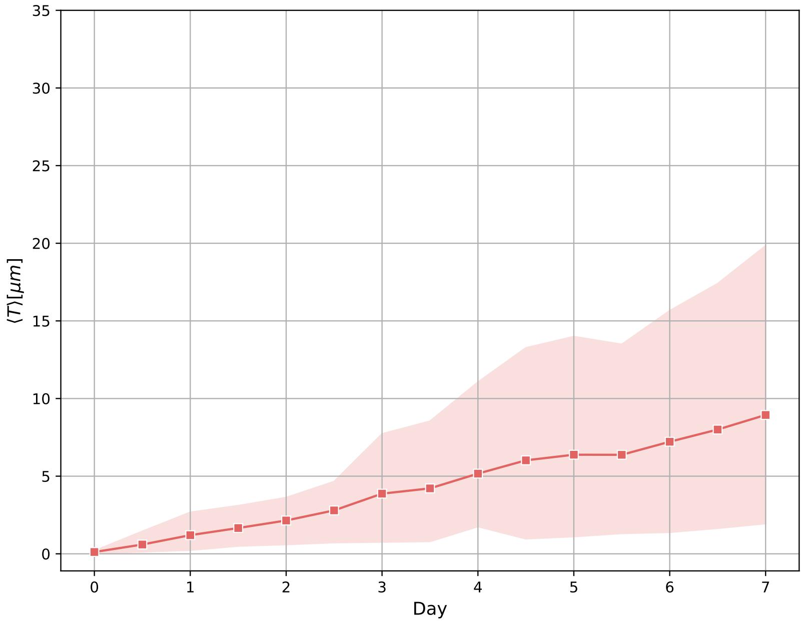

Supplementary Figure 18. Case 4, $\tau_w = 0.27\,\mathrm{Pa}$

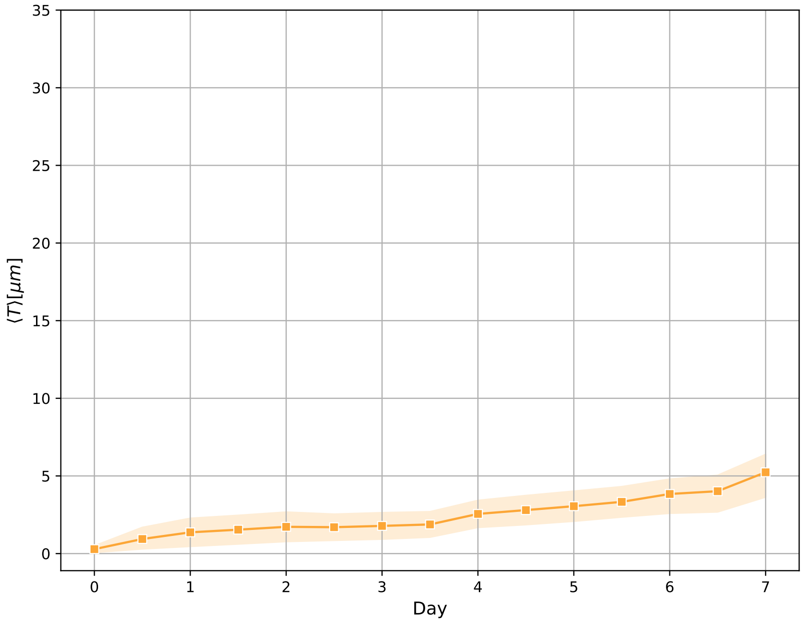

Supplementary Figure 19. Case 5, $\tau_w = 0.34\,\mathrm{Pa}$

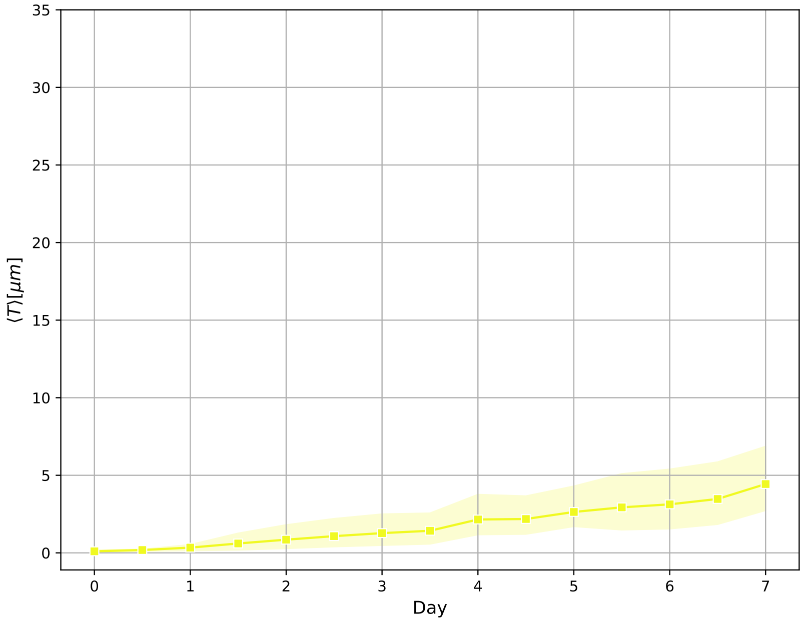

Supplementary Figure 20. Case 6, $\tau_w = 0.67\,\mathrm{Pa}$
